# Prognostic factors for survival and radiation necrosis after stereotactic radiosurgery alone or in combination with whole brain radiation therapy for 1–3 cerebral metastases

**DOI:** 10.1186/1748-717X-9-105

**Published:** 2014-05-02

**Authors:** Lars Hendrik Schüttrumpf, Maximilian Niyazi, Silke Birgit Nachbichler, Farkhad Manapov, Nathalie Jansen, Axel Siefert, Claus Belka

**Affiliations:** 1 Department of Radiation Oncology, University of Munich, Marchioninistr 15, Munich 81377, Germany; 2 Department of Nuclear Medicine, University of Munich, Marchioninistr 15, Munich 81377, Germany; 3 Gemeinschaftspraxis für Strahlentherapie und Radioonkologie am Klinikum Schwabing, Kölner Platz 1, Munich 80804, Germany

**Keywords:** Stereotactic radiotherapy, Cerebral metastases, Radiosurgery, Radiation necrosis

## Abstract

**Background:**

In the present study factors affecting survival and toxicity in cerebral metastasized patients treated with stereotactic radiosurgery (SRS) were analyzed with special focus on radiation necrosis.

**Patients and methods:**

340 patients with 1–3 cerebral metastases having been treated with SRS were retrospectively analyzed. Radiation necrosis was diagnosed by MRI und PET imaging. Univariate and multivariate analysis using a Cox proportional hazards regression model and log-rank test were performed to determine the prognostic value of treatment-related and individual factors for outcome and SRS-related complications.

**Results:**

Median overall survival was 282 days and median follow-up 721 days. 44% of patients received WBRT during the course of disease. Concerning univariate analysis a significant difference in overall survival was found for Karnofsky Performance Status (KPS ≤ 70: 122 days; KPS > 70: 342 days), for RPA (recursive partitioning analysis) class (RPA class I: 1800 days; RPA class II: 281 days; RPA class III: 130 days), irradiated volume (≤2.5 ml: 354 days; > 2.5 ml: 234 days), prescribed dose (≤18 Gy: 235 days; > 18 Gy: 351 days), gender (male: 235 days; female: 327 days) and whole brain radiotherapy (+WBRT: 341 days/-WBRT: 231 days). In multivariate analysis significance was confirmed for KPS, RPA class and gender. MRI and clinical symptoms suggested radiation necrosis in 21 patients after SRS +/− whole brain radiotherapy (WBRT). In five patients clinically relevant radiation necrosis was confirmed by PET imaging.

**Conclusions:**

SRS alone or in combination with WBRT represents a feasible option as initial treatment for patients with brain metastases; however a significant subset of patients may develop neurological complications. Performance status, RPA class and gender were identified to predict improved survival in cerebral metastasized patients.

## Introduction

Cerebral metastases are diagnosed in about 30% of patients with advanced tumors [1,2]. Lung cancer, breast cancer and malignant melanoma are the most common causes for brain metastases. Symptoms depend on localization and size including signs of increased intracranial pressure, headaches, vertigo, nausea and vomiting, paraesthesia and seizures.

Patients having more than three brain metastases are generally treated with whole-brain radiotherapy (WBRT). Oligometastatic patients with 1–3 lesions have a better prognosis and are therefore treated more aggressively. Beside neurosurgical resection stereotactic radiosurgery (SRS) is an effective treatment option for patients with 1–3 brain metastases [3,4]. For radiation treatment some studies have shown that SRS alone might be superior to WBRT alone for survival advantage of RPA class I patients [5,6]. It cannot be excluded that this effect is partially caused by the available salvage options after radiosurgery.

In three randomized trials additional WBRT showed even better intracranial tumor control and reduced neurologic causes of death but failed to improve patients overall survival and functional independence [7-9]. The 1-year local control rates at the initial tumor site after neurosurgical resection or SRS +/− WBRT were about 80% [3,4,7,8]. Intracranial relapse occurred more frequently in patients having received SRS or resection only. In this context WBRT was used more often as a salvage treatment. The deferred WBRT probably improved the length of the survival and functional independence in the observation arm. The latest Cochrane Analysis of WBRT reported an improved local and distant brain control but no difference in overall survival for SRS + WBRT compared to SRS alone [10].

SRS as well as WBRT has a risk for adverse events. Radiation necrosis appears 1–2 years after radiotherapy (RT) and cognitive decline develops over many years. For fractionated RT (<2.5 Gy/d) high cumulative doses are tolerated. Radiation induced necrosis is predicted to occur in 5% at a biologically effective dose of 120 Gy [11]. For SRS a correlation between the target volume, dose and the risk of adverse events has been demonstrated [12,13]. However the tolerated doses for SRS show a great range in literature. In dose escalation study RTOG 90–05 maximum tolerated doses were 24 Gy, 18 Gy, and 15 Gy for tumors ≤ 20 mm, 21–30 mm, and 31–40 mm [14].

The present study was performed to assess factors that have prognostic relevance on survival in cerebral metastasized patients treated with stereotactic radiosurgery and to assess side effects with a special focus on radiation induced necrosis.

## Patients and methods

### Patient data and dose fractionation

Between March 2000 and December 2010 340 patients with 1–3 cerebral metastases were treated with stereotactic radiosurgery.

Patients with stable systemic disease at the time of SRS or general cerebral progression during follow-up received additional WBRT. The prescribed dose for WBRT usually was 35 Gy/37.5 Gy in 14/15 fractions of 2.5 Gy or 30 Gy in 10 fractions of 3 Gy at midline, 5 fractions per week. Patients showing further single brain metastases during follow-up, but stable systemic disease, again were treated with SRS.

### Head frames

For stereotactic radiosurgery a Brown-Robert-Wells (BRW) or Gill-Thomas-Cosman (GTC) stereotactic head frame was used. While the BRW frame is fixated to the head with four screws to ensure a definite connection between cranium and head frame the GTC frame is less invasive by using dental fixation. Afterwards a planning computed tomography (CT) with localizer was performed. To ensure the correct position of the head frame a depth helmet was used to measure the distance between cranium and surface of the helmet. This control was done before planning CT and immediately before stereotactic radiosurgery.

### Radiation planning

For radiation planning and image fusion Radionics Xknife™ was used. The gross tumor volume (GTV) was identified and delineated in fused image of the CT and the magnetic resonance imaging (MRI). Due to spherical growth of brain metastases the clinical target volume (CTV) was set equivalent to the GTV. Regarding risk structures and anatomical borders expansion of the GTV plus 1–2 mm resulted in the planning target volume (PTV).

### Dose

Radiation dose was 24 Gy for metastases with a diameter <20 mm, 18 Gy for metastases between 20–30 mm and 15 Gy for a diameter >30 mm prescribed to the 80% isodose. Due to the geometry of the metastases some patients received 20 Gy. A modified Linear Accelerator (Mevatron M/Fa. Siemens) with 6 MV photons was used for treatment. The PTV was irradiated with three to eight arcs depending on size and localization.

### MRI protocol

A T1 weighted contrast enhanced sequence was used to determine the gross tumor volume. No image tilt (0°) was allowed. Slice thickness ≤ 3 mm, Inter-Slice-Spacing 0 mm.

### FET-PET

For PET scan the amino acid [^18^ F]-fluoro-ethyl-L-tyrosine (FET) was used. FET uptake in the tissue was measured as standardized uptake value (SUV). Maximum lesion-to-brain ratios (LBRs) were calculated and time–activity curves were analyzed.

### Statistics

The patient data was collected between March 2000 and December 2010. All analyses were performed using the Statistical Package for Social Sciences (SPSS, Ver. 19.0, SPSS Inc, Chicago, IL). Survival analyses were based on Kaplan-Meier estimates, univariate testing was performed by means of the log-rank test and Cox regression analysis was used to determine hazard ratios as well as to perform a multivariate analysis. A two-tailed p-value ≤ 0.05 was considered significant.

### Follow-up and assessment of radiation necrosis

Follow-up was regularly performed every three months. If patients did not keep the appointments, a telephone follow-up was used. Cerebral staging was done by contrast enhanced MRI. If radiation necrosis was suspected, diagnostics was completed either by FET-PET and/or brain biopsy, or patients were treated with dexamethasone only ex juvantibus. Alternatively a control MRI was done about 4 weeks later.

## Results

### Patient characteristics

Between March 2000 and December 2010 340 patients (159 males and 181 females) with newly diagnosed brain metastases were treated with SRS (Table 1). The median age was 62 years. One hundred ninety seven patients (57.9%) had one metastasis, 101 patients (29.7%) had two metastases and 31 patients (9.1%) had three metastases. Some patients showing further single brain metastases but stable systemic disease during follow-up were again treated with SRS. Therefore eight patients had four metastases, two patients had five metastases and one patient even had seven metastases in total. The most common tumor types were lung cancer (55%), malignant melanoma (14.4%) and breast cancer (12.1%). More than half of the patients (55.6%) received no additional WBRT. Referring to the Radiation Therapy Oncology Group (RTOG) based recursive partitioning analysis (RPA) 26 patients (7.7%) were RPA class I, 271 patients (79.7%) RPA class II and 43 patients (12.6%) RPA class III. Two hundred fifty eight patients (75.9%) had a Karnofsky Performance Score > 70.

**Table 1:** Baseline patient characteristics, N = 340, WBRT – whole brain radiotherapy

**Characteristic**	**Patients**
Median follow-up	721 days
Sex	
• Male	159 (46.8%)
• Female	181 (53.2%)
Median age	62
Age	
• < 65 y	208 (61.2%)
• ≥ 65 y	132 (38.8%)
WBRT	
• No	189 (55.6%)
• Yes	151 (44.4%)
RPA class	
• I	26 (7.7%)
• II	271 (79.7%)
• III	43 (12.6%)
Karnofsky Performance Score	
• ≤ 70	82 (24.1%)
• > 70	258 (75.9%)
Number of cerebral metastases	
• 1	197 (57.9%)
• 2	101 (29.7%)
• 3	31 (9.1%)
• 4	8 (2.4%)
• 5	2 (0.6%)
• 7	1 (0.3%)
Histology	
• NSCLC	160 (47.1%)
• SCLC	27 (7.9%)
• Malignant Melanoma	49 (14.4%)
• Breast Cancer	41 (12.1%)
• Renal Cell Carcinoma	20 (5.9%)
• Others	43 (12.6%)
Median radiation volume	1.7 ml
Median dose to the 80% isodose	20 Gy
Radiation necrosis	
• Yes	21 (6.2%)
• No	319 (93.8%)

### Survival data

**Figure 1:**
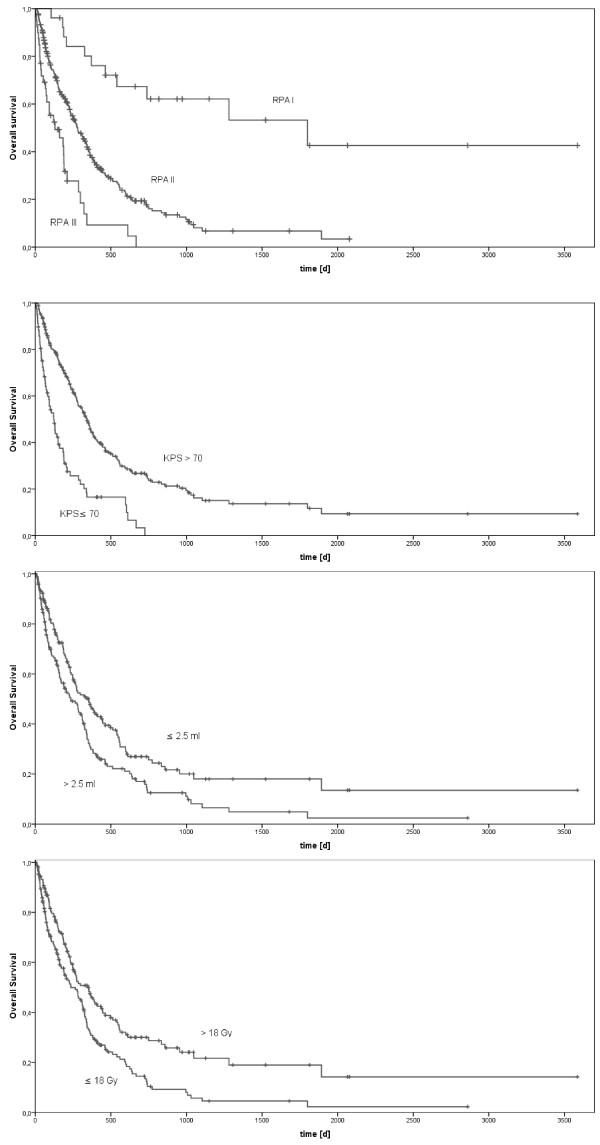
Kaplan-Meier analysis of overall survival for RPA classes (p < 0.001), KPS (p < 0.001), volume (p = 0.002) and dose (p < 0.001).

The median follow-up of all patients was 721 days using the reverse Kaplan-Meier [15]. The median survival time after SRS was 282 days; 1-year survival rate was 28.8% and 2-year survival rate was 10.6%. Survival data after stereotactic radiosurgery of the cerebral metastases is shown in Table 2. Univariate analysis of potential prognostic factors showed a significant difference for Karnofsky Performance Status (KPS ≤ 70: 122 days; KPS > 70: 342 days; p < 0.001), for RPA (recursive partitioning analysis) class (RPA class I: 1800 days; RPA class II: 281 days; RPA class III: 130 days; p < 0.001), irradiated volume (≤2.5 ml: 354 days; > 2.5 ml: 234 days; p = 0.002), prescribed dose (≤18 Gy: 235 days; > 18 Gy: 351 days; p < 0.001), gender (male: 235 days; female: 327 days; p = 0.013) and whole brain radiotherapy (+WBRT: 341 days/-WBRT: 231 days; p = 0.049) (Figure 1). For categorical analysis of volume and dose dependent overall survival the largest lesion in first irradiation session was used to categorize patients having more than one metastasis (median volume 2.5 ml and median dose 18 Gy for dominant lesion).

**Table 2:** Univariate and multivariate analysis on potential prognostic factors for overall survival after stereotactic radiosurgery, ns – not significant, CI – confidence interval

**Factor**	**Number of patients**	**Median survival time in days**	**Univariate analysis**		**Multivariate analysis**	
			**95%-CI in days**	**p-value (unadjusted, log-rank)**	**p-value (adjusted, cox-regression)**	**Hazard ratio**
Sex				0.013	0.042	1.31
• Male	159	235	172 – 298			
• Female	181	327	274 – 380			
• Overall	340	282	232 – 332			
WBRT				0.049	ns (0.067)	1.28
• No	189	231	172 – 290			
• Yes	151	341	280 – 402			
• Overall	340	282	232 – 332			
KPS				< 0.001	< 0.001	2.19
• ≤ 70	82	122	84 – 160			
• > 70	258	342	298 – 386			
• Overall	340	282	232 – 332			
Histology				ns (0.488)		
• NSCLC	160	275	218 – 332			
• SCLC	27	231	151 – 331			
• Melanoma	49	286	201 – 371			
• Breast Ca	41	383	153 – 613			
• RCC	20	184	0 – 435			
• Others	43	265	36 – 582			
• Overall	340	282	217 – 494			
Age				ns (0.1)		
• < 65 y	208	306	254 – 358			
• ≥ 65 y	132	247	177 – 317			
• Overall	340	282	232 – 332			
Number of metastases				ns (0.764)		
• 1	197	268	203 – 333			
• 2	101	275	183 – 367			
• 3	31	332	0 – 710			
• 4	8	467	47 – 905			
• 5	2	341	–			
• 7	1	834	–			
• Overall	340	282	232 – 332			
Dose				< 0.001	ns	
• ≤ 18 Gy	175	235	156 – 314			
• > 18 Gy	165	351	255 – 447			
	340	282	232 – 332			
Volume				0.002	ns	
• ≤ 2.5 ml	172	354	262 – 446			
• > 2.5 ml	168	234	162 – 306			
• Overall	340	282	232 – 332			
RPA class				< 0.001		
• I	26	1800	362 – 3238		< 0.001	
• II	271	281	227 – 335		< 0.001	3.61
• III	43	130	17 – 243		0.001	3.85
• Overall	340	282				

Multivariate analysis revealed the following factors to be statistically significant predictors for survival: the Karnofsky Performance Status (hazard ratio (HR) 2.19 for KPS ≤ 70 vs. KPS > 70; p < 0.001), RPA (HR 3.61 for RPA II vs. I, HR 3.85 for RPA III vs. I; p < 0.001 for RPA I, p < 0.001 for RPA II, p = 0.001 for RPA III) and gender (HR 1.31 for male vs. female; p = 0.042) (Table 2).

### Analysis of radiation necrosis

**Figure 2:**
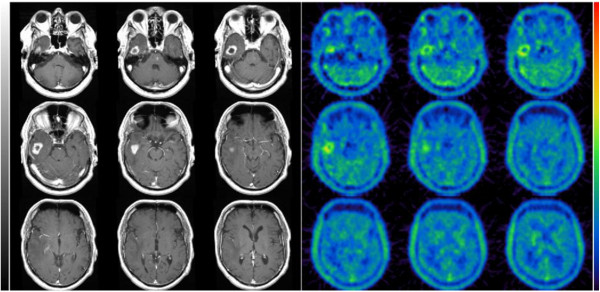
**Example of recurrent brain metastasis: the study shows pathologic contrast enhancement on T1-weighted MRI and corresponding increased [**^**18**^ **F]-FET uptake.** While radiation necrosis was suspected in MRI, PET showed a pathologic FET uptake as seen in recurrent metastasis.

Radiation necrosis is regarded as the most relevant adverse event after SRS. During follow-up in 21 patients (6.2%) radiation necrosis was supposed in MRI. The most common neurologic symptom was dizziness. Two patients had cerebellar ataxia. Eleven patients received FET-PET to differentiate between radiation necrosis und recurrent metastasis (Figure 2). The maximum lesion-to-brain ratios (LBRs) were higher in patients with recurrent metastases (n = 6; mean LBR = 2.55) than in patients with radiation necrosis (n = 5; mean LBR = 1.84). Time–activity curves were assessed in nine patients with ten cerebral lesions. In five patients early peak of FET uptake followed by constant decline of uptake was seen (decreasing kinetics). These were all patients with high LBRs and were assumed to have recurrent metastasis. Four of these five patients had brain biopsy or neurosurgical resection. In two cases recurrent metastasis was confirmed histologically, the other patients had necrosis only. Dynamic evaluation of four patients with low LBRs showed constantly increasing FET uptake until the end of acquisition confirming radiation necrosis. Volume (p = 0.151), prescribed dose (p = 0.236) and fractionated WBRT (p = 0.368) had no influence on radiation necrosis.

## Discussion

The current study was performed to define prognostic factors for survival and the incidence of radiation necrosis in cerebral metastasized patients after treatment with stereotactic radiosurgery. The median overall survival was 282 days. Different prognostic factors could be identified including Karnofsky Performance Status, RPA class, irradiation volume, prescribed dose, gender and additional WBRT. Despite WBRT and gender these factors are well known to correlate with survival prognosis. RPA classes which were created as a prognostic tool are defined by age, KPS, presence of extracranial metastases and control of primary tumor. The overall survival of patients in the study cohort was 59.2 months for RPA class I, 9.2 months for RPA class II and 4.3 months for RPA class III. Compared to other historical groups the survival time, especially for RPA class I, is rather high. Sneed et al. reported a median survival time after SRS alone or SRS + WBRT of 14 and 15.2 months for RPA class I, 8.2 and 7 months for RPA class II and 5.3 and 5.5 for RPA class III [16]. In our study cohort only a few patients (7.6%) met criteria for RPA class I and had more favorable features.

Male patients had a significantly shortened overall survival compared to females. This could be explained by the fact that fewer women smoke than men and breast cancer, which has better prognosis per se, occurred in women exclusively. Breast cancer as histologic entity had no significantly improved overall survival. This could be explained by the limited number of cases.

In categorical analysis high dose (>18 Gy) and small irradiation volume (≤2.5 ml) correlated with prolonged survival. Both parameters might result in an increased local control rate with a decreased chance for neurological cause of death.

The univariate analysis of survival data suggested a significant survival benefit for patients that had received a whole-brain radiotherapy (SRS + WBRT: 341 days/SRS alone: 231 days; p = 0.049). Another retrospective analysis was associated with a trend towards improved survival for additional WBRT (median survival time 15.4 versus 8.3 months, p = 0.08) [17].

None of the prospective randomized studies could confirm these findings: Adding postoperative WBRT after surgical resection of single metastases prevented brain recurrence of tumor (18% vs 70%, p < 0.001) and reduced neurologic cause of death (14% vs 44%, p = 0.003) compared to patients in the observation group. There was no significant difference between the 2 groups in overall length of survival or the length of time that patients remained functionally independent [9].

In another randomized trial from Japan 132 patients with 1 to 4 brain metastases where either treated with SRS + WBRT (65 patients) or with SRS alone (67 patients). The median survival time and 1-year survival rate were 8.0 months and 28% for the SRS group and 7.5 months and 39% for the SRS + WBRT group (p = 0.42). The 1-year local control rate (73% vs. 89%, p = 0.003) and 1-year distant control rate (36% vs. 58%, p = 0.003) were better for the combined treatment [7].

The latest prospective trial with 359 patients was published in 2011 by Kocher et al. 199 patients received SRS, 160 patients were treated with surgical resection. After SRS, 100 patients were allocated to the observation group, 99 were allocated to WBRT. After surgery, 81 patients received WBRT while 79 patients had no further treatment. The median overall survival time, including surgical patients, was 10.7 months for the observation group and 10.9 for WBRT group (p = 0.89). The 2-year local control rate (69% vs. 81%, p = 0.04) and 2-year distant control rate (52% vs. 67%, p = 0.023) were improved by WBRT. Death caused by intracranial progression was 44% in the observation group and 28% in the WBRT group. Salvage therapies, e.g. WBRT, had to be used more frequently in the observation group [8]. Regarding the health-related quality-of-life no sustained decline in physical, role, and cognitive functioning were found. The latest randomized trial described transient changes in quality-of-life only [18].

In the retrospective analysis of the data base no difference was made between WBRT as an initial or salvage treatment. Given the fact that patients with favorable histology and stable extracranial disease were not distributed equally a selection bias cannot be excluded. Under these circumstances the survival benefit for WBRT has to be judged cautiously. Local control rates were not documented.

The most common late toxicity for SRS is radiation necrosis. In 21 patients (6.2%) radiation necrosis after SRS was assumed in MRI. Radiation necrosis can be difficult to distinguish from tumor recurrence on MRI and may require the use of surgery, positron emission tomography (PET) or magnetic resonance spectroscopy (MRS). In patients having neurologic symptoms maximum SUV and dynamic evaluation of FET-PET confirmed radiation necrosis in five individuals. Additional WBRT had no influence on the occurrence of radiation necrosis. Due to low incidence in the study no predictive factors for radiation necrosis were found. While the risk of radiation necrosis after conventional radiotherapy is highest in the first 2 years after treatment, appearance of radiation necrosis after SRS can be as short as 3 months [19]. In literature the incidence of brain necrosis varies from about 5 – 32% [7,8,13,20-22]. Higher rate of necrosis occur with longer follow-up [14]. By using the pattern of time–activity curve in FET-PET local brain metastasis recurrence can be differentiated from radiation necrosis with high accuracy [23]. Radiation dose, tumor volume and radiation treatment planning factors are predictive for radiation induced necrosis [12,24,25]. In RTOG 90–05 study tumor volume > 8.2 ml and a ratio of maximum dose to prescription dose > 2 were significantly associated with unacceptable toxicity [26]. The only predictive parameter influencing the risk of radiation necrosis described by Valery et al. was the conformity index [27].

## Conclusions

SRS alone or in combination with WBRT is an effective treatment for patients with 1–3 cerebral metastases; however a small subset of patients may develop neurological complications. KPS and RPA class are relevant prognostic factors for overall survival. Especially for patients with favorable features additional WBRT should be considered without having increased rates of necrosis.

## Consent

Written informed consent was obtained from the patient for the publication of this report and accompanying images.

## Abbreviations

BG: Background; FET: [^18^ F]-fluoro-ethyl-L-tyrosine; HR: Hazard ratio; Ns: Not significant; NSCLC: Non-small-cell lung cancer; KPS: Karnofsky performance status; MM: Malignant melanoma; MRI: Magnetic resonance imaging; PET: Positron emission tomography; PTV: Planning target volume; RCC: Renal cell cancer; RPA: Recursive partitioning analysis; SCLC: Small-cell lung cancer; SRS: Stereotactic radiosurgery; SUVmax: Maximum standardized uptake value; vs: Versus; WBRT: Whole brain radiotherapy.

## Competing interests

The authors declare that they have no competing interests.

## Authors’ contributions

LHS collected patient data and wrote the manuscript. MN performed associated statistics, designed the protocol and critically revised the manuscript. AS built the departmental database and collected patient data. NJ analyzed FET-PET. SBN, AS and FM critically revised the manuscript, too. CB provided the idea and conception and took part in the preparation of the manuscript. All authors read and approved the final manuscript.
